# Insulinoma Management in a Pregnant Woman: A Case Report

**DOI:** 10.7759/cureus.34239

**Published:** 2023-01-26

**Authors:** Bertha Patricia Diaz-Sangines, Julio Gonzalez-Cofrades, Eric Emilio Vazquez-Camacho, Montserrat Malfavon-Farias, Linda Garcia-Lima

**Affiliations:** 1 Gynecology, Centro Médico ABC, Mexico City, MEX

**Keywords:** complications, fistula, pregnancy, hypoglycemia, insulinoma

## Abstract

An insulinoma is a rare tumor that originates in the β cells of the pancreas with an incidence of four cases per million people per year. To our knowledge, 40 cases of insulinoma in pregnancy have been reported. Insulinoma in pregnancy is usually diagnosed in the first trimester. Surgical treatment is advised during the second trimester with complications arising in up to 10% of cases. We present the case of a 34-year-old pregnant woman diagnosed with insulinoma during the first trimester. The patient underwent a laparoscopic resection of the tumor at 15.5 weeks of gestation. Insulinoma was resected and postoperative complications were managed, including acute pancreatitis and peripancreatic and splenorenal fluid collection, which were treated using multidisciplinary management and resolved at the end of the pregnancy at 40.1 weeks of gestation.

## Introduction

An insulinoma is a rare tumor of the endocrine pancreas that originates in the cells of the ductal/acinar system. This tumor produces high amounts of insulin and causes dysregulation of the serum glucose, resulting in clinical manifestations, such as preprandial or postprandial hypoglycemia. Although the incidence of insulinoma is extremely low in the general population at around four cases per million people per year [1], it is the most common neuroendocrine tumor of the pancreas. The average age of onset is 50 to 60 years, with a higher incidence in women (59%) [1]. Diagnosis is often a challenge due to its low occurrence, particularly in pregnant women, as many symptoms of insulinoma are similar to those of pregnancy, including hunger, diaphoresis, anxiety, palpitations, and nausea. Other neuroglycopenic symptoms include headache, lethargy, dizziness, diplopia, amnesia, confusion, and, in severe cases, seizures and coma [2-4]. Currently, only 40 cases of insulinoma in pregnant women have been reported worldwide, with only one case reported in Mexico [5]. Most cases are recognized or become symptomatic during the first trimester [6-8].

Current diagnosis and therapeutic approaches for suspected insulinoma in pregnancy raise several concerns. First, a prolonged supervised fasting test should be done in pregnant patients with hypoglycemia and insulinemia, which is the most important endocrinological examination for the diagnosis of insulinoma. However, this test is invasive for both the mother and the fetus. Second, imaging studies such as computed tomography (CT), magnetic resonance imaging (MRI) with contrast agents, and endoscopic ultrasound, which are essential for localization and staging of insulinoma, can pose some risk to the fetus or mother, making this tumor difficult to detect during pregnancy [7,9]. The treatment of choice for insulinoma is the same as in non-pregnant patients. Surgical resection is the preferred option, along with preoperative treatment with diazoxide or octreotide [4,7]. In this article, to our knowledge, we present the second case of gestational insulinoma reported in Mexico. We also review the literature on its diagnosis and management.

## Case presentation

A 34-year-old patient with a relevant medical history of controlled hypothyroidism presented to the prenatal care visit at 10 weeks of gestation of a singleton pregnancy. The patient reported fainting episodes in the last four years, which increased in frequency and duration during pregnancy until the symptoms were daily. These fainting episodes were previously treated with topiramate without improvement. Laboratory tests detected a glycemic serum level of 30 mg/dL with an oral glucose tolerance curve of 25 mg/dL at two hours. A multidisciplinary team evaluated the patient, who corroborated the hypoglycemic levels, and detected a proinsulin level of 197.7 pmol/L (0-10 pmol/L). An abdominal ultrasound and MRI revealed no pancreatic lesions. As her symptoms persisted, a glycemic test was performed and again showed hypoglycemia (30 mg/dL) without insulin suppression and a proinsulin level of 380 pmol/L. Endoscopic ultrasound revealed a lesion of 6 × 10 mm within the body of the pancreas, which was suspected to be an insulinoma.

At 15.5 weeks of gestation, the patient was admitted for surgical pancreatic exploration and laparoscopic enucleation of a mass measuring 1 × 0.5 cm. On the second postoperative day, she started to develop oral intolerance, abdominal pain, and increased peripancreatic drainage output. An abdominal ultrasound revealed imaging compatible with pancreatitis. Her lipase level was 54 U/L, indicating mild edematous acute pancreatitis with pancreatic fistula. Parenteral nutrition, octreotide, meropenem, and two units of red blood cells, due to anemia, were administered. She was discharged 19 days later without any alarming obstetric data and with pancreatic fluid collection and surgical abdominal drainage.

**Figure 1:**
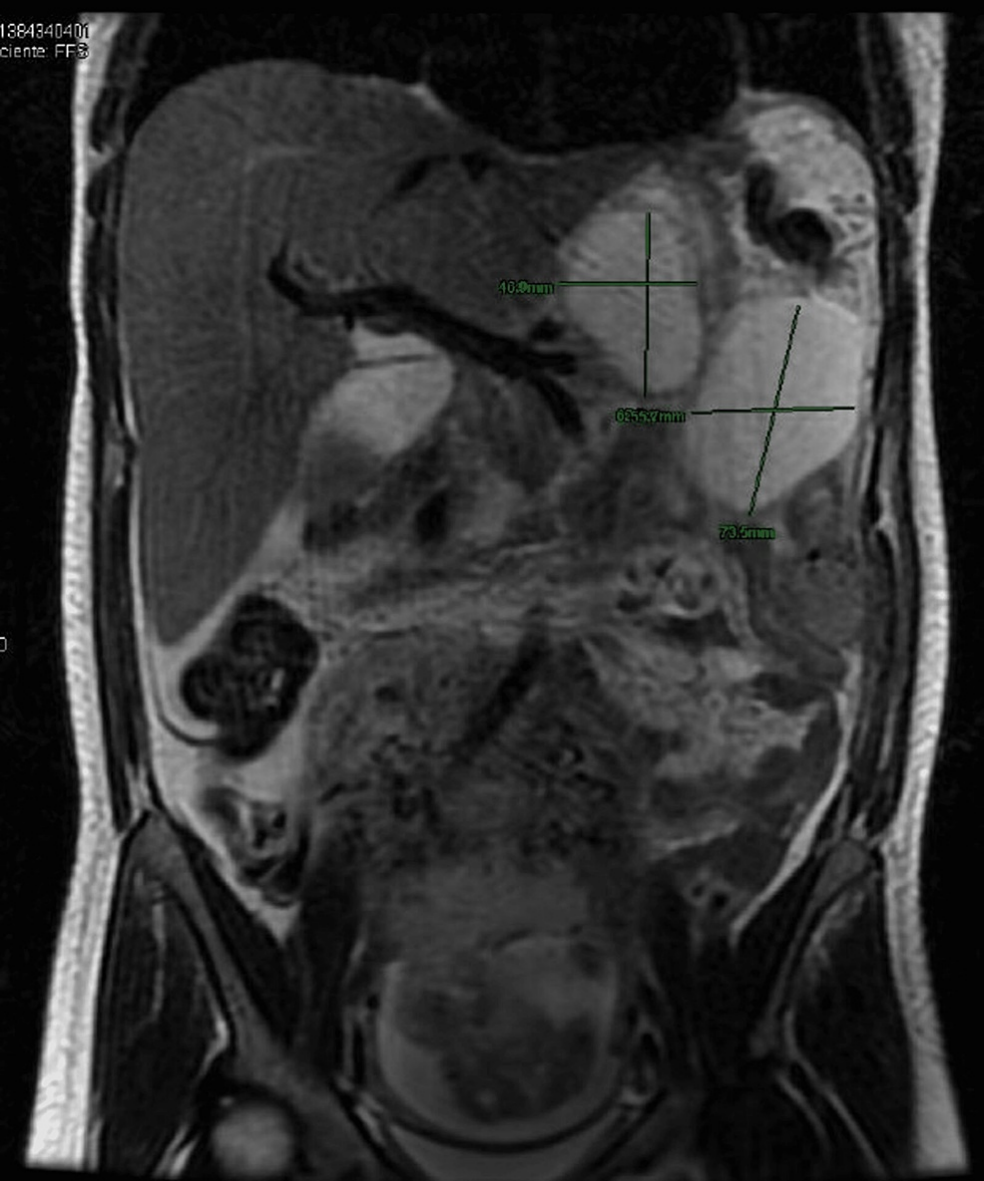
Magnetic resonance imaging of the abdomen showing peripancreatic fluid collection and splenorenal collection in the left hypochondrium.

She was readmitted at 22 weeks of gestation due to persistent oral intolerance and epigastric pain. An MRI of the abdomen showed peripancreatic and splenorenal fluid collection (Figure 1). Endoscopic drainage was performed. The patient was observed for 17 days, during which her general condition and oral tolerance improved. MRI showed a decrease in the size of the splenorenal and peripancreatic fluid collections. Therefore, the patient was discharged.

The patient was admitted to the hospital for a third time at 29.3 weeks of gestation for oral intolerance. General tests and a follow-up MRI showed no abdominal abnormalities; however, there was a threat of preterm labor which was managed with uterine inhibitors (nifedipine and orciprenaline) and betamethasone to ensure pulmonary maturation. A nasogastric tube was placed to ensure an adequate response.

After one week, the patient was discharged and remained without symptoms and comorbidities for 10 weeks. Her nasogastric tube remained in place during this time, and she was tolerating a per-oral diet, with a weight gain of 7 kg during the third trimester. At 40.1 weeks of gestation, a female baby was born via cesarean section. The baby weighed 3,038 g, with an APGAR score of 9/10 and no evidence of hypoglycemia or hyperinsulinemia. The patient showed adequate evolution, her glucose levels remained within normal parameters, and the puerperal phase concluded without complications.

## Discussion

Insulinomas are a rare pathology, and very few cases of insulinoma in pregnancy have been reported in the literature [4-8,10-12]. These tumors are characterized by symptomatic hypoglycemia, based on the results of a fasting glucose test at 48 to 72 hours [7,13], with neuroglycopenic symptoms. The diagnosis of insulinoma is made when elevated levels of serum insulin are present in a spontaneous or induced episode of hypoglycemia. Insulin levels greater than 6 mU/L with glucose levels less than or equal to 45 mg/dL, together with a C-peptide level greater than or equal to 0.2 nmol/L, negative sulfonylurea, and negative anti-insulin antibodies, indicate the presence of an insulinoma [5,13]. Most (87%) insulinomas are singular and benign, 7% are multiple and benign, and 6% are malignant and present with metastasis [1,14]. Little is known about the malignant potential of these tumors. However, they are indolent with a long-term survival rate, even in cases that present with metastasis [14].

Diagnosis can be particularly difficult in pregnancy because although hypoglycemia is rare in non-diabetic pregnant patients, it can occur in diabetic patients who are on insulin treatment [7]. Signs of hypoglycemia typically appear in the first 16 weeks of gestation and, though rare, can occur immediately after childbirth in the presence of Sheehan’s syndrome due to severe postpartum hemorrhage. The Whipple triad is used to diagnose an episode of hypoglycemia. It includes the presence of neuroglycopenic symptoms, low levels of glucose serum, and the correction of these symptoms following the normalization of glycemic levels. The triad can serve as a guide for the diagnosis of insulinoma [15]. Moreover, many other symptoms of insulinomas are common in pregnancy, such as hypotension, dizziness, and nausea, especially in the early stages of gestation. As pregnancy progresses, circulating glucose levels in the mother increase to maintain the glucose supply to the fetus. Secretion of hormones such as human placental lactogen and tumor necrosis factor-alpha are also secreted from the placenta [16,17].

Insulinomas have an average size between 0.2 and 2 cm and are equally distributed throughout the pancreas [18]. Several imaging techniques (e.g., transabdominal ultrasound, endoscopic ultrasound, CT, MRI, and intra-arterial calcium stimulation test) can identify precise locations [4,13]. Ultrasound is a widely available, non-invasive, and inexpensive tool that allows the evaluation of the pancreas without radiation exposure. However, it is highly operator dependent and has limited depth. Endoscopic ultrasound has proven to be the most accurate technique for diagnosis and localization, with a sensitivity and specificity of 95%, allowing the detection of smaller pancreatic tumors, as in this case report. Although CT and MRI are not operator dependent, they depend on the protocol and the machine used to provide precise and detailed diagnostic information, allowing the detection of very small abnormalities; however, both CT and intra-arterial calcium stimulation testing are associated with high radiation exposure [4].

Treatment for symptoms of hypoglycemia includes dietary management, diazoxide, calcium channel blockers, and octreotide. Due to the limited number of patients treated with octreotide during pregnancy, the possible benefits of octreotide treatment must be carefully weighed against its possible risks [4]. Medical treatment may be considered in cases when symptomatic hypoglycemia in pregnancy is easy to control or if the patient refuses surgery. However, surgery is the only curative treatment for insulinoma, with a 77-100% cure rate [19]. Laparoscopic procedures including enucleation of the insulinoma, partial distal pancreatectomy, Whipple procedure, and total pancreatectomy have comparable success rates in pregnant and non-pregnant populations, with minimal mortality and strong safety profiles. Such procedures are recommended during the second trimester between 12 and 17 weeks of gestation. The laparoscopic approach is usually preferred when the tumor is well localized and small, with a complication rate of approximately 10% [2,19,20].

## Conclusions

In this case of a 34-year-old pregnant patient, the insulinoma was detected within the first 12 weeks of gestation and had the best possible prognosis. A multidisciplinary team was able to treat the patient with a favorable outcome for both the mother and the baby. A surgical procedure was chosen due to the patient’s poor glycemic control, which placed her in the category of high-risk pregnancy due to possible complications. It is believed that the key to the success in this case was close surveillance, with the timely detection and proper management of complications, as well as the follow-up provided by the team of surgeons, gastroenterologists, endocrinologists, nutritionists, and gynecologists.

## References

[1] Service FJ, McMahon MM, O'Brien PC, Ballard DJ (1991). Functioning insulinoma--incidence, recurrence, and long-term survival of patients: a 60-year study. Mayo Clin Proc.

[2] Vaidakis D, Karoubalis J, Pappa T, Piaditis G, Zografos GN (2010). Pancreatic insulinoma: current issues and trends. Hepatobiliary Pancreat Dis Int.

[3] Grant CS (2005). Insulinoma. Best Pract Res Clin Gastroenterol.

[4] Besemer B, Müssig K (2010). Insulinoma in pregnancy. Exp Clin Endocrinol Diabetes.

[5] Viruez-Soto J antonio, Vallejo-Narváez CM, Torrez-Morales F (2014). Insulinoma y embarazo. Caso de medicina crítica en ginecología y obstetricia. Gaceta Mexicana Oncol.

[6] Takacs CA, Krivak TC, Napolitano PG (2002). Insulinoma in pregnancy: a case report and review of the literature. Obstet Gynecol Surv.

[7] Christiansen E, Vestergaard H (2008). Insulinoma in a third-trimester pregnant woman combined with pre-eclampsia: a case report and review of the diagnostic strategies. Gynecol Endocrinol.

[8] Dobrindt EM, Mogl M, Goretzki PE, Pratschke J, Dukaczewska AK (2021). Insulinoma in pregnancy (a case presentation and systematic review of the literature). Rare Tumors.

[9] Abe T, Takeda Y, Takiyama T (2021). A case of insulinoma diagnosed postpartum with hypoglycemic symptoms that were masked during pregnancy. Clin Case Rep.

[10] Diaz AG, Herrera J, López M, Puchulu FM, Ferraina P, Bruno OD (2008). Insulinoma associated with pregnancy. Fertil Steril.

[11] de Albuquerque Neto CC, da Silva Lira N, Albuquerque MA, Santa-Cruz F, de França M Vasconcelos L, Ferraz ÁAB, Costa AC (2019). Surgical resection of pancreatic insulinoma during pregnancy: case report and literature review. Int J Surg Case Rep.

[12] Tomazic M, Janez A, Ravnik Oblak M (2017). Hypoglycemia identified by a continuous glucose monitoring system in a second-trimester pregnant woman with insulinoma: a case report. J Med Case Rep.

[13] De León DD, Stanley CA (2013). Determination of insulin for the diagnosis of hyperinsulinemic hypoglycemia. Best Pract Res Clin Endocrinol Metab.

[14] Maggio I, Mollica V, Brighi N, Lamberti G, Manuzzi L, Ricci AD, Campana D (2020). The functioning side of the pancreas: a review on insulinomas. J Endocrinol Invest.

[15] Whipple A (1938). The surgical therapy of hyperinsulinism. J Int Chir.

[16] Baeyens L, Hindi S, Sorenson RL, German MS (2016). β-Cell adaptation in pregnancy. Diabetes Obes Metab.

[17] Rodrigues Queiróz AJ, Nazareno LS, Miranda JE (2012). Insulinoma diagnosed in the postpartum: clinical and immunohistochemical features. Gynecol Endocrinol.

[18] Jensen RT, Berna MJ, Bingham DB, Norton JA (2008). Inherited pancreatic endocrine tumor syndromes: advances in molecular pathogenesis, diagnosis, management, and controversies. Cancer.

[19] Thakker RV, Newey PJ, Walls GV (2012). Clinical practice guidelines for multiple endocrine neoplasia type 1 (MEN1). J Clin Endocrinol Metab.

[20] Cogliandolo A, Pidoto RR, Causse X, Kerdraon R, Saint Marc O (2001). Minimally invasive management of insulinomas. A case report. Surg Endosc.

